# Research progress on the role and mechanism of miR-671 in bone metabolism and bone-related diseases

**DOI:** 10.3389/fonc.2022.1018308

**Published:** 2023-01-11

**Authors:** Shaotai Wang, Min Hu, Dongsheng Song, Linjun Tang, Huan Jiang

**Affiliations:** ^1^ Department of Orthodontics, Hospital of Stomatology, Jilin University, Changchun, China; ^2^ Jilin Provincial Key Laboratory of Tooth Development and Bone Remodeling, Changchun, China

**Keywords:** bone metabolism, miR-671, lncRNA, inflammation, cancer

## Abstract

Bone metabolism consists of bone formation and resorption and maintains a dynamic balance *in vivo*. When bone homeostasis is broken, it can manifest as osteoarthritis (OA), rheumatoid arthritis (RA), osteosarcoma (OS), etc. MiR-671, an important class of non-coding nucleotide sequences *in vivo*, is regulated by lncRNA and regulates bone metabolism balance by regulating downstream target proteins and activating various signaling pathways. Based on the structure and primary function of miR-671, this paper summarizes the effect and mechanism of miR-671 in bone-related inflammation and cancer diseases, and prospects the application possibility of miR-671, providing reference information for targeted therapy of bone-related disorders.

## Introduction

Bone metabolism includes bone formation and bone resorption. Osteoblasts and osteoclasts are essential cells in maintaining bone homeostasis. Bone components maintain dynamic balance by regulating cytokines and metal ions in the body. When there is exogenous or endogenous stimulation, abnormal metabolism of osteoblasts and osteoclasts can be manifested as osteoarthritis (OA), rheumatoid arthritis (RA), osteosarcoma (OS), etc. MicroRNAs (miRNAs) are non-coding RNAs ranging from 18 to 25 nucleotides in length, which play an essential role in biological processes such as cell proliferation, differentiation, migration, apoptosis, and tumorigenesis. MiRNAs can also regulate signaling pathways in the cytoskeleton, inflammation, and metabolism. MiRNAs have gradually become biomarkers for diagnosing and prognostic evaluating many diseases (1). With continuous research, microRNA-671 (miR-671) has been found to reflect the degree of inflammation and tumorigenesis in bone metabolism and related disorders. Its influence on bone metabolism and bone-related diseases has been gradually clarified. This paper mainly reviews the impact of miR-671 on bone metabolism and the occurrence and development of bone-related diseases to make early diagnosis and targeted therapy based on the changes of miR-671 content *in vivo* in clinical practice.

## Structure and basic functions of miR-671

**Figure 1:**
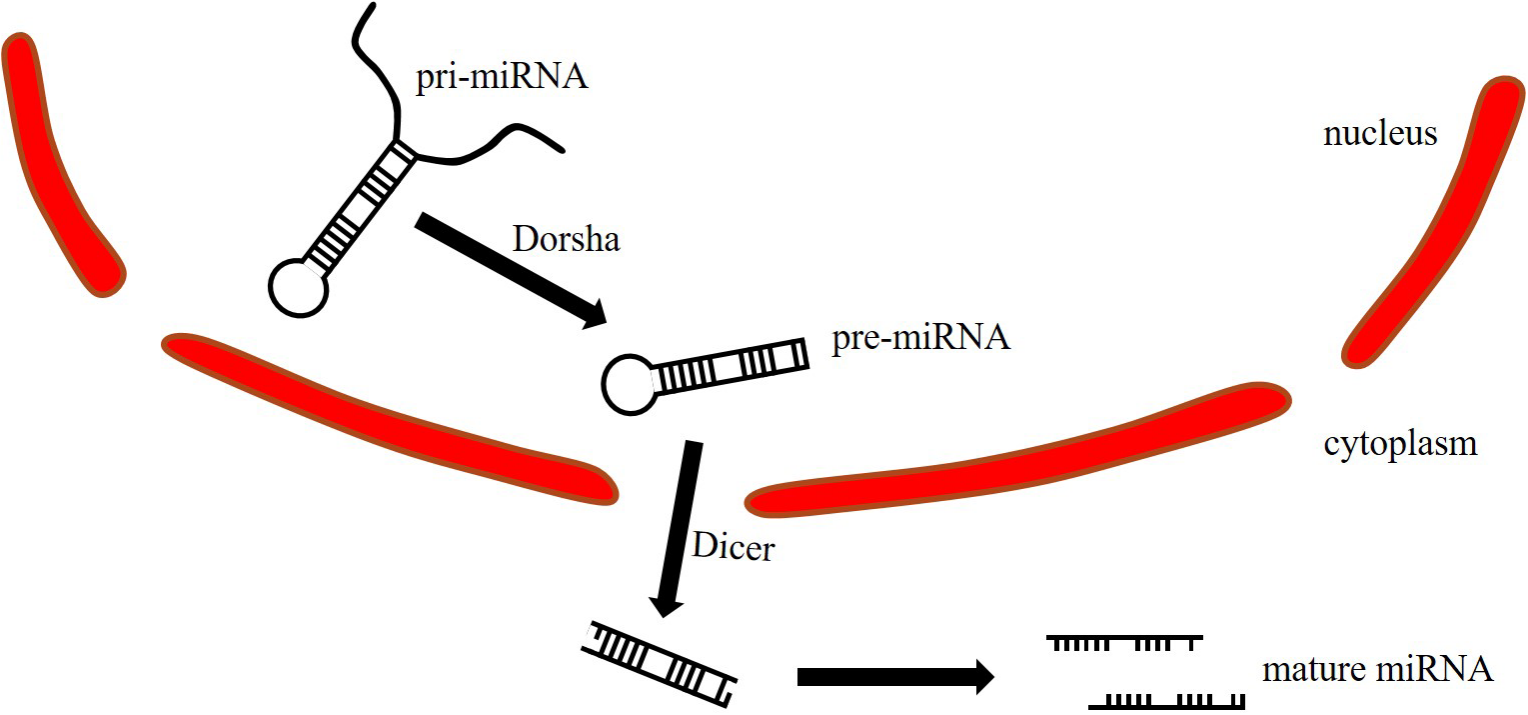
miRNA maturation process.

MiRNA maturation begins with the primary miRNA (pri-miRNA) in the nucleus, and the length is between 300 and 10,000 bp (**Figure 1**). Pri-miRNA is cleaved by Dorsha to generate precursor miRNA (pre-miRNA). The pre-miRNA is 70-90bp in length and transferred from the nucleus to the cytoplasm. Then, the Dicer opens the hairpin structure of the pre-miRNA to generate double-stranded miRNA, which is further cleaved to generate two single-stranded miRNAs (2). The passenger strand is removed and degraded, while the guide strand is retained to form the RNA-induced silencing complex (RISC) (3). The guide strand is stable under Argonaute (AGO) protein protection from cellular exoribonucleases (4, 5). MiRNA regulates gene expression by fully or incompletely binding to the 3’UTR seed sequence of the target gene mRNA, resulting in degradation or translation (6).

**Figure 2:**
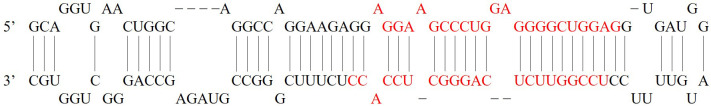
pre-miR-671 stem-loop structure (7). The red sequence is the mature miRNA. miR-671-5p: 29-AGGAAGCCCUGGAGGGGCUGGAG-51. miR-671-3p: 68-UCCGGUUCUCAGGGCUCCACC-88.

**Figure 3:**
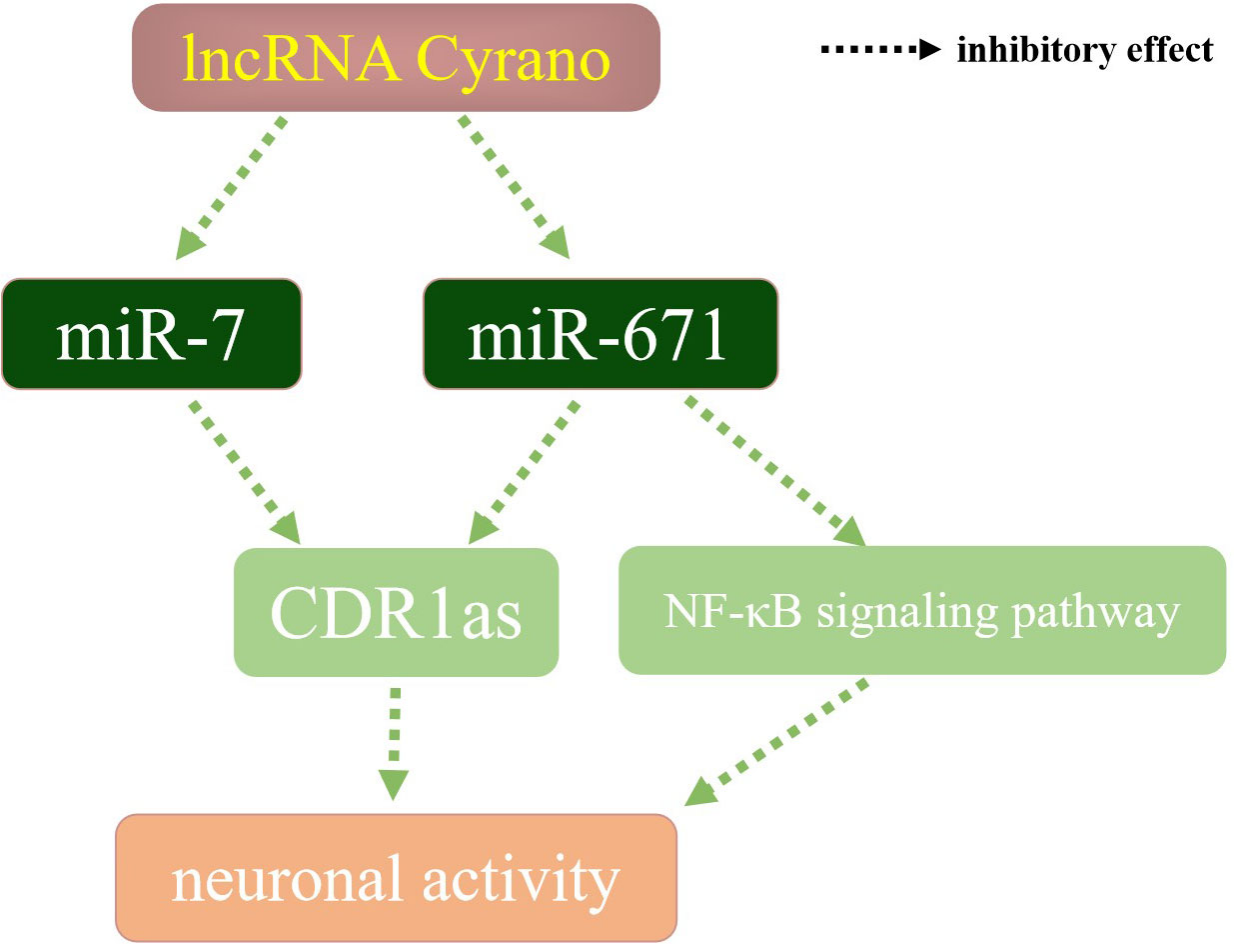
miR-671 in nervous system regulation.

As miRNA can be derived from different stem arms, pre-miR-671 generates two miRNAs, miR-671-5p and miR-671-3p (**Figure 2**), respectively, both of which play an essential role in the stable regulation of the human body environment by mainly binding to the target mRNA (8). Content change of components in the lncRNA-miRNA-mRNA axis could affect the downstream pathways, such as TGF-β signaling (9) and Wnt/β-catenin signaling (10). In terms of nervous system regulation (**Figure 3**), miRNA-671 can affect the content of mRNA and circRNA in mammalian brain signal networks (11) and regulates neuronal apoptosis with lncRNA and circRNA (12). MiR-7 is one of the first known and also most investigated miRNAs. It is considered a network stabilizer and connects different signaling pathways, especially in the central nervous system (13, 14). LncRNA Cyrano can degrade miR-7 through a wide range of pairing sites, thereby increasing the content of CDR1as and negatively regulating neuronal activity. When the content of lncRNA Cyrano is decreased, miR-7 and miR-671 will increase. MiR-7 and miR-671 inhibit downstream CDR1as together (15, 16), thereby increasing neuronal activity. Reduced expression of miRNA-671-5p has been detected in Parkinson’s disease, ischemic stroke, and other diseases (17, 18), which weakens the inhibitory effect of NF-κB, and the macroscopic manifestations are increasing neuroinflammatory responses. MiR-671 also plays a vital role in maintaining the dynamic balance of bone metabolism. Studies on the osteogenic differentiation of dental pulp stem cells and bone marrow stem cells showed that miR-671-3p and miR-671-5p expressions were up-regulated (19). When bone metabolism is abnormal and the bone microenvironment is destroyed, the content of miR-671 changes in varying degrees.

## MiR-671 in bone-related inflammatory diseases

When inflammation occurs *in vivo*, miR-671-5p, as a critical miRNA, can affect the expression of inflammatory genes. It can be used as a biomarker to diagnose inflammation, such as Kawasaki disease and hepatitis (20–23), reflecting the degree of inflammation and affecting the healing time of injury (24). Inflammation is often related to the development and progression of cancer. Inflammation-related cells are genetically stable and do not rapidly develop drug resistance and invasiveness (25). As inflammation evolves, repeated stimulation affects normal cells, leading to genetic changes and increasing cancer risk, similar to bone inflammatory diseases. Therefore, to diagnose and treat bone inflammatory diseases early and reduce the risk of canceration, the content of miR-671 can be detected to clarify the process of relevant inflammatory reactions.

### Mechanisms of miR-671 regulating the inflammatory response

Inflammation will increase the permeability of local blood vessels, and liquid and cellular components exudate. It releases many cytokines and increases the number of white blood cells. Also, inflammation-related factors can affect cell functions through miRNA (26). The effective elimination of pathogens depends on several signaling pathways, such as Toll-like-receptor (TLR) signaling; many of these are regulated by miRNA (27). MiR-671, as a biomarker, is involved in macrophage immune signal transduction in the early stage of the inflammatory response (28–30). Activated macrophages release various immune regulatory factors and reduce the expression of miR-671 (31).

Significant up-regulation of miR-671-5p can be detected in both periodontitis and late stage of hepatitis B, thus inhibiting inflammatory response (22, 23). The relative fold change significantly increased in advanced fibrosis versus early fibrosis. MiR-671-5p can also act as a target to mediate immune regulation, affect cytokine production, and changes in antigen-related marker levels (32). Meanwhile, miR-671-3p exists in the miRNA-mRNA-immune cell regulatory network (33). It can be a biomarker for predicting and diagnosing grades II-IV acute graft-versus-host disease (34). MiR-671-3p RQ level<0.5 (down expressed) could also be observed when inflammation is reducing (35). Therefore, miR-671 level is usually up-regulated during the middle and late stages of inflammatory responses. Thus, there is a positive correlation between the miR-671 level and inflammation.

### Mechanisms of miR-671 regulating the bone-related inflammatory diseases

OA is a degenerative joint disease characterized by articular cartilage degeneration and inflammation. Many studies have confirmed that miR-671 is closely related to OA. Through miRNA sequencing analysis of tissues, plasma, and chondrocytes of OA patients, the results showed that miR-671-3p level was down-regulated during the rapid progression of the disease (36), and the expression of the downstream target gene, TNF receptor-associated factor, was enhanced (37). In the late stage of the disease, the increase of miR-671 can reduce the inhibition of cell proliferation and apoptotic stimulation induced by IL-1β and reduce the expression of type II collagen in chondrocytes, which can reduce the progression of OA (38). Therefore, miR-671-3p reflects the progressive state of bone metabolism and can be used as a potential biomarker to assess the risk and progression of OA (39). The circRNA-miRNA-mRNA regulatory network plays a crucial role in the regulation of OA (40). Upstream circRNA usually acts as a miRNA sponge in cells and weakens the inhibitory effect of miR-671 on inflammation through the competitive binding of miR-671. Compared with healthy people, the expression of circ-IQGAP1 and circ_0114876 increases in cartilage tissues, while the expression of miR-671-5p decreases (41). Reducing circRNA content can reduce chondrocyte apoptosis, inflammatory cytokine secretion, and extracellular matrix degradation induced by IL-1β.

**Figure 4:**
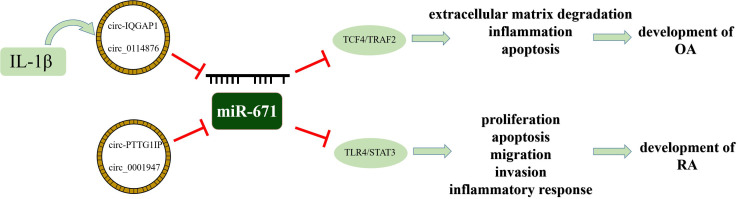
The schematic diagram of the involvement of the circRNA-miRNA-mRNA axis in OA and RA development.

In terms of regulating RA, circRNA pituitary tumor-transforming 1 interacting protein (circ-PTTG1IP) regulates toll-like receptor 4 (TLR4), an upstream regulator of the nuclear factor-kappa B (NF-κB), by directly binding to miR-671-5p. CircRNAs could act as a miRNA sponge to absorb the functional miRNAs, such as miR-671-5p, which could suppress the function of TLR4. If the expression of circRNA is increased, the proliferation, migration, and invasion of RA-flss can be promoted by activating the NF-κB pathway, and local inflammatory response can be affected (42). Similarly, the upstream target of miR-671-5p, circ_0001947, can also up-regulate the expression of downstream target STAT3 by absorbing miR-671-5p, thus promoting the progression of RA (43) (**Figure 4**). Therefore, drugs for RA can reduce the expression of circRNA by influencing the circRNA-miRNA-mRNA regulatory network, thus enhancing the expression of miR-671-5p, inhibiting the inflammatory response, and promoting RA healing (44).

## MiR-671 and OS

Persistent inflammation can lead to cell damage and fibrosis, increasing the risk of canceration (45). Cytogenetic changes often accompany the generation of cancer. MiRNAs participate in post-transcriptional regulation and interfere with oncogenes or tumor suppressor genes by binding to the 3′UTR region of mRNA, thus affecting cancer development (46).

OS is a highly invasive bone tumor that occurs mainly in young patients. Like most cancers, miR-671, as a tumor suppressor gene, plays a significant role in OS. The expression of miR-671-5p is significantly decreased in tissues and cells of patients with OS. Overexpression of miR-671-5p inhibits the proliferation, migration, and invasion of OS cells. MiR-671-5p directly binds cyclin D1 and CDC34 to inhibit cell cycle progression, thus inhibiting the progression of cancer (47). TUFT1 and SMAD3, the targets of miR-671-5p, can enhance the migration and invasion of OS cells. Low expression of miR-671-5p and high expression of TUFT1 and SMAD3 will lead to poor prognosis (48, 49). LncRNA DLEU1, an upstream regulator of miR-671, is highly expressed in the tissues and cells of patients with OS. Through directly absorbing miR-671-5p, lncRNA DLEU1 regulates the expression of DEAD-box helicase 5 and plays an oncogenic role in OS (50). Therefore, by targeting the upstream and downstream genes of miR-671-5p, the regulation of the lncRNA-miRNA-mRNA network can be a targeted therapy for OS.

**Figure 5:**
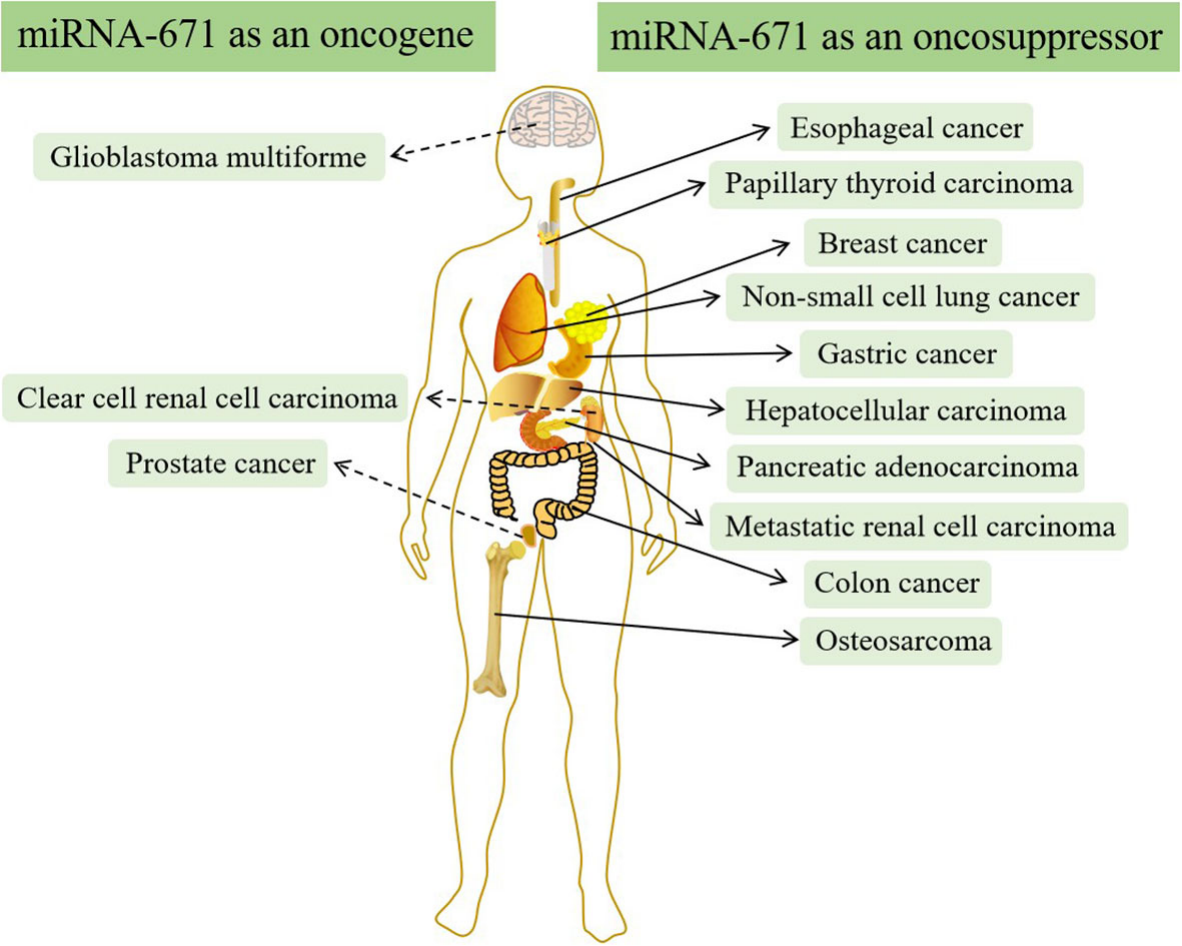
miRNA-671 in different types of cancer.

In addition to OS, there is increasing evidence that miR-671 is often abnormally expressed in other types of cancer and can regulate the structure and expression of mRNA and protein (51–53). LncRNA-miRNA-mRNA regulatory network plays a significant role in cancer’s occurrence, development, and prognosis by regulating downstream target genes and proteins (54–57). Therefore, targeted therapy drugs can inhibit cancer growth by influencing the regulatory network (58, 59). In most types of cancer, such as breast cancer, esophageal cancer, non-small cell lung cancer, hepatocellular carcinoma, etc., miR-671 acts as a tumor suppressor and is down-regulated in cancer (60–69). However, in a few kinds of cancer, miR-671 acts as an oncogene and is up-regulated *in vivo (* 70–77) (**Figure 5**).

## Conclusion

Abnormal bone metabolism affects bone quality and leads to an increased incidence of fractures. The continuous exploration of miR-671 in clinical and basic experiments will promote the application of miR-671 in the screening and treatment of abnormal bone metabolism more and more widely. In this review, we collect the studies on miR-671 in recent years and summarize the role of miR-671 in bone metabolism and bone-related diseases. However, there are few studies on the mechanism of miR-671 regulating bone metabolism through various signaling pathways. In the future, searching for critical genes and using miRNA-targeted therapy can block the development of the diseases by studying the pathogenesis. Thus, doctors can reduce the cost of treatment and the damage of inflammation and cancer.

## Author contributions 

SW, DS and LT contributed to the research retrieval and drafting. MH and HJ contributed to the outline drafting and critically revised the manuscript. Every author gave final approval and agreed to be accountable for all aspects of the work. All authors have read and agreed to the published version of the manuscript. All authors contributed to the article and approved the submitted version.
